# Corrosion Failure Mechanism of Associated Gas Transmission Pipeline

**DOI:** 10.3390/ma11101935

**Published:** 2018-10-11

**Authors:** Weimin Zhao, Timing Zhang, Yonglin Wang, Jianhua Qiao, Zerui Wang

**Affiliations:** School of Materials Science and Engineering, China University of Petroleum, Qingdao 266580, China; zhangtm@upc.edu.cn (T.Z.); m13045013792@163.com (Y.W.); 15610036410@163.com (J.Q.); m13455289561@163.com (Z.W.)

**Keywords:** associated gas, corrosion, failure mechanism, gas pipeline

## Abstract

Corrosion has been responsible for several gas pipeline leakage accidents; thus, clarifying its failure mechanisms is a precondition to prevent such accidents. On the basis of failure analysis of corroded pipe sections, laboratory exposure tests were conducted by simulating three possible corrosion environments inside a gas pipeline. The corrosion rate indicated by depth change was adopted in this study. Scanning electron microscopy and X-ray diffraction were used to analyze corrosion products. Results showed that the specimens completely immersed in condensate water were generally corroded and that the specimens exposed to gas were locally corroded. However, the corrosion rate of the latter was slightly lower; hence, no autocatalysis of occluded corrosion cell occurred in the formation of corrosion pit, and uniform corrosion occurred in the precipitation location of condensate water. The areas in the range of 5 mm below the waterline indicated severe corrosion, and the rate could reach twice that of other areas. The corrosion products were mainly FeO(OH) and FeCO_3_, thereby proving that the corrosion failure of pipelines was caused by oxygen absorption corrosion and CO_2_ corrosion. Suggestions were presented to control corrosion failure of associated gas pipelines.

## 1. Introduction

Associated gas is the gas that is obtained in crude oil production; the main ingredients of such associated gas are methane, ethane, and hydrocarbons [1]. In the absence of a gas market, the traditional means of managing associated gas are reinjection, flaring, and venting [2]. The flaring or venting of associated gas not only causes energy waste but also adversely affects the environment. In contrast, reinjection is a type of treatment method that is beneficial to sustainable development [3,4,5]. In some oil fields, associated gas is used as a lift gas to raise oil recovery [6]. Gas lifting is the process of injecting compressed natural gas (called lift gas) into the production tubing of an oil well, and the lift gas increases the pressure gradient between the reservoir and well fluid that pushes the fluid to the surface [7]. Usually, the compressed gas from a field processing facility is routed to oil gathering manifold and wellhead via pipelines.

Associated gas in many cases has not been subjected to strict dehydration treatment, which can be classified as wet gas. The wet gas contains liquid hydrocarbon and water, acid gases (such as CO_2_ and H_2_S), and dissolved ions (such as Cl^−^ and Ca^2+^) [8,9]. Under the combined action of temperature, pressure, and stress, serious corrosion occurs in the inner wall of steel pipes [8,10,11]. Internal corrosion is the main factor that affects the safety of natural gas pipelines [12,13,14]. It can reduce the thickness of thick walls, reduce the strength of pipelines, and lead to leakage accidents. Once the gas transmission pipeline leaks, it not only results in considerable economic loss but also causes environmental pollution. Therefore, studying the corrosion failure mechanism of gas transmission pipelines is necessary to propose control measures.

Thus far, many studies have been conducted on corrosion and its control of gas pipelines. Most researchers have deemed that the presence of water and dissolved gases, such as CO_2_ and H_2_S, introduce serious internal corrosion [12,15,16,17,18]. Many factors that promote liquid collection, including gas quality, operating parameters, and pipeline features, affect the likelihood of internal corrosion [19]. The water phase that wets the steel surface determines the initiation of corrosion [16]. CO_2_ corrosion is considered the main cause of corrosion in natural gas pipelines [20,21], and the corrosion rate is accelerated by O_2_ [22], H_2_S and organic acids [23,24], and salt content [25,26]. Various laboratory tests have been conducted to study the corrosion of steel pipelines under full immersion in the water or gas phase under water condensing conditions [17,26]. However, the liquid depth in gas pipelines is relatively small in most cases, and the corrosion law of steel pipelines under thin liquid film and in large-volume liquid should be different. No research has shown the corrosion of the liquid–gas transition zone near a waterline. In addition, CO_2_ corrosion is not necessarily the root cause of gas pipeline corrosion. The corrosion mechanism of gas pipelines changes with gas impurities. At present, corrosion research on CO_2_ and O_2_ coexistence conditions remains insufficient because corrosion products may change with changes in their partial pressure.

In this study, the corrosion mechanism of associated gas pipelines was developed for actual engineering failure accidents. Gas lift facilities were commissioned in 2009–2010, and associated gas was used as the lift gas. The associated gas contained water, CO_2_ and O_2_. Several leaks were observed in the associated gas lines in November 2014. Then, the situation became increasingly serious, thereby seriously affecting the normal operation of pipelines. Relevant research is urgently needed to determine the root cause of the leak and take remedial measures. Therefore, the corrosion failure mechanism of the associated gas pipeline was clarified by simulating three corrosion environments inside a gas pipeline on the basis of observing corrosion morphology and analyzing corrosion products.

## 2. Materials and Methods

### 2.1. Description of the Pipeline

**Figure 1:**
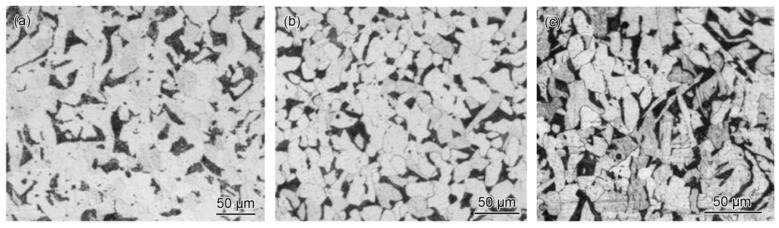
Microstructures of pipes with different diameters: (**a**) 2″, (**b**) 4″, and (**c**) 6″.

The investigated pipelines are buried trunk lines (151.1 mm × 7.1 mm, 6″ pipe) and flow lines (102.3 mm × 6.0 mm, 4″ pipe; 50.7 mm × 4.8 mm, 2″ pipe) for conveying the associated gas. The pipe material is ANSI B31.8 API 5L X42. The chemical compositions of the collected pipe materials were determined by a Metallab 75/80 direct-reading spectrometer (GNR, Novara, Italy), and the results are listed in Table 1. The tensile properties of the collected pipe materials were investigated by a WDW-300E computer-controlled universal testing machine (Shijin Testing Equipment, Jinan, China), and the test results are shown in Table 2. The corresponding acceptance criteria are also listed in Table 1 and Table 2 as Spec. It can be seen from Table 1 and Table 2 that the chemical compositions and tensile properties of the three pipes with different diameters are all acceptable based on the requirements for PSL 2 seamless X42 pipe in API Spec 5L-2013; however, the content of sulfur in the 2″ pipe is more than the requirement. The microstructures of the 2″, 4″ and 6″ steel pipes in cross section, longitudinal section, and inner surface layer were observed by a Leica DM2500 M optical microscope (Leica Microsystems, Wetzlar, Germany). No essential difference was found between cross-sectional, longitudinal-sectional, and inner surface microstructures; therefore, only the microstructures in the cross-section are shown in Figure 1. It is obvious that the microstructures are normal and are composed of ferrite and pearlite. 

The compositions of representative gas samples are listed in Table 3. The main ingredients of the gas are hydrocarbon (methane, ethane, propane, butane, pentane, and >C5), nitrogen, CO_2_, and oxygen. Furthermore, 1.2–2.5 mg/kg H_2_S is present in the gas. The ion concentration of representative condensate water is listed in Table 4. The main cations are Na^+^, K^+^, Ca^2+^, Li^+^, and Mg^2+^. The main anions are SO_4_^2-^ and Cl^-^. The pH value of condensate water is 6.65. The operating pressure is approximately 11 MPa, and the temperature is in the range 41–45 °C. The normal flow is 3500, 38,400, and 120,000 m^3^/d in the 2″, 4″, and 6″ pipelines, with corresponding flow rates of 1.1, 5.9, and 12.2 m/s, respectively.

### 2.2. Laboratory Studies of Corroded Pipe Sections

The information from the site investigation indicates that most leakage points are located at the bottom of the pipeline, whereas some are located on the side; only a few are located at the top. Representative corroded pipe sections were collected for laboratory studies. The internal corrosion morphology was observed macroscopically by a camera and microscopically by using a JXA 8230 scanning electron microscope (SEM, JEOL, Tokyo, Japan). The chemical composition of corrosion products on the pipe surface of the 2″, 4″, and 6″ steel pipe was obtained by energy-dispersive spectroscopy (EDS) analysis using the JXA 8230 SEM. The phase compositions of corrosion products were analyzed using an X’ Pert PRO MPD X-ray diffraction (XRD, PANalytical B.V., Almelo, The Netherlands).

### 2.3. Corrosion Simulation Tests

New pipe sections, which have the same pipe material with the leaking pipe, were collected. Steel specimens were machined from the pipe sections. The dimensions of specimens from the 2″ steel pipe are 40 × 13 × 2 mm^3^, and those from the 4″ and 6″ steel pipes are 40 × 13 × 3 mm^3^. All the specimens were ground to 2000-grade emery paper and cleaned with distilled water and absolute ethyl alcohol.

Corrosion simulation tests were conducted following ASTM G111 “Standard guide for corrosion tests in high-temperature or high-pressure environment, or both”. The tests were conducted in a kettle, and the specimens were positioned in three different environments. One-third of the total specimens was totally immersed in the water phase, which is a condition that corresponds to the so-called “bottom of the line” situation in gas pipelines; another one-third of the total specimens was partly immersed in the solution, which represents the working environment of the part of the pipe that corresponds to the transfer zone from liquid to gas environment; and the final one-third of the total specimens was exposed to high-pressure gas phase under water condensing conditions, which corresponds to the “top of the line” situation. The gas and the solution were prepared based on Table 3 and Table 4, and nitrogen was used to replace the organic components in the simulated gas. Four specimens were investigated for each pipe in each environment; one of them was used for SEM observation and XRD analysis, and the other three were used to calculate the corrosion rate. The test temperature was 43 °C, and the test pressure was 11 MPa. The corrosion tests lasted 135 h.

The corrosion rates with different corrosion failure modes were determined in various ways. The weight loss rate was used to evaluate the general corrosion rate, and the depth of local corrosion pits was measured by Leica DM2500 M confocal microscopy. For comparison, the rate of depth change was used to represent the corrosion rate. Corrosion products were analyzed by EDS and XRD.

## 3. Results

### 3.1. Macroscopic Observation of Corroded Pipe Sections

**Figure 2:**
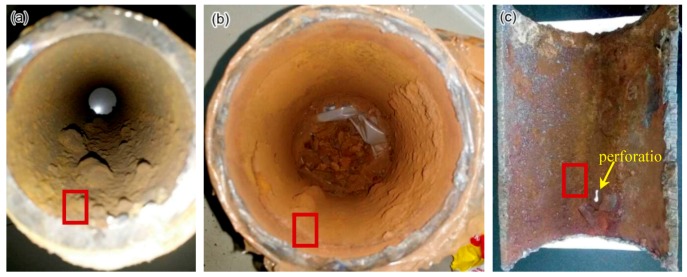
Corrosion morphology on the internal walls of steel pipe sections: (**a**) 2″ pipe, (**b**) 4″ pipe and (**c**) 6″ pipe.

Figure 2 shows the typical corrosion morphology on the internal walls of the 2″, 4″, and 6″ steel pipe sections with internal corrosion. The upper and lower positions of the pipe section in Figure 2a,b are consistent with the working status. Corrosion products are distributed across all the exposed surfaces. However, the corrosion products on the bottom part of pipe sections, especially in pipes with a small diameter, are thick and nonuniform. A perforation occurs on the bottom part of the 6″ pipe section (Figure 2c), and thickness reduction can be clearly observed around the perforation, thereby indicating that the perforation is induced by internal corrosion. The corrosion environment for the bottom part of the pipe sections is different from those for the side and top parts, and the bottom parts are likely covered with condensate liquid.

### 3.2. Analysis of Corrosion Products

**Figure 3:**
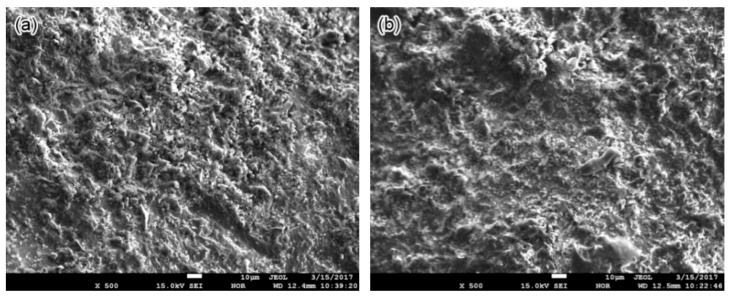
Top view of corrosion scales on the 2″ pipe: (**a**) flat area and (**b**) raised area.

**Figure 4:**
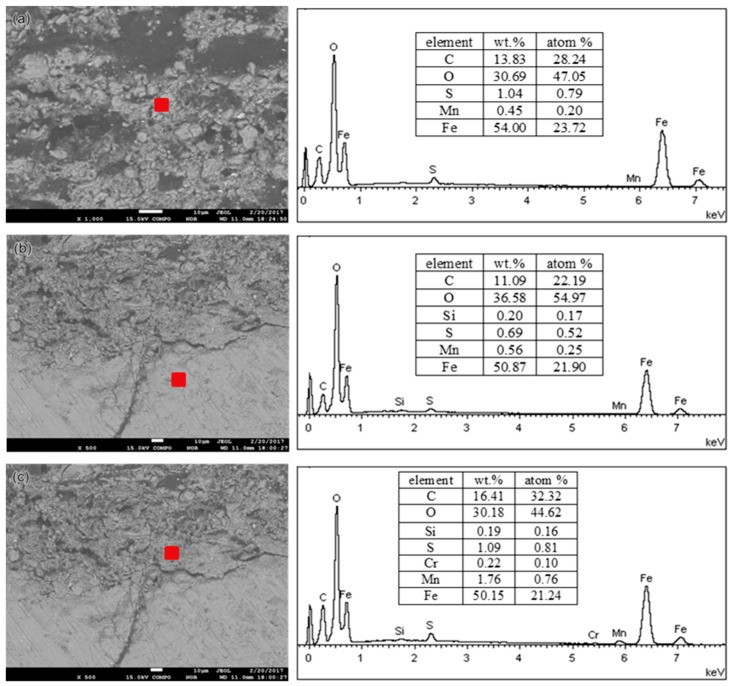
EDS analysis results of corrosion products on the 6″pipe: (**a**) surface, (**b**) inner layer and (**c**) outer layer.

An SEM equipped with EDS was applied to investigate the cross-sectional and surface morphology and chemical compositions of corrosion products on the pipe surface of the 2″, 4″, and 6″ steel pipe sections. The approximate observed positions are marked in Figure 2 with red boxes. No obvious micromorphology differences occur between smooth scale (Figure 3a) and raised corrosion products (Figure 3b). Subsequently, the chemical composition of corrosion products on the 2″, 4″, and 6″ steel pipes were analyzed by EDS. The results show that no essential difference is found in the composition of scales along its thickness direction for different pipes. Therefore, only the analysis results on the 6″ steel pipe are presented in Figure 4. The main elements include Fe, C and O. Then, the corrosion products are speculated to be oxides and carbonates from the reaction between steel and O_2_ and CO_2_ in the environment. Furthermore, few S is found in corrosion products, and the material should be sulfide of Fe, which is the reaction product of steel with H_2_S.

**Figure 5:**
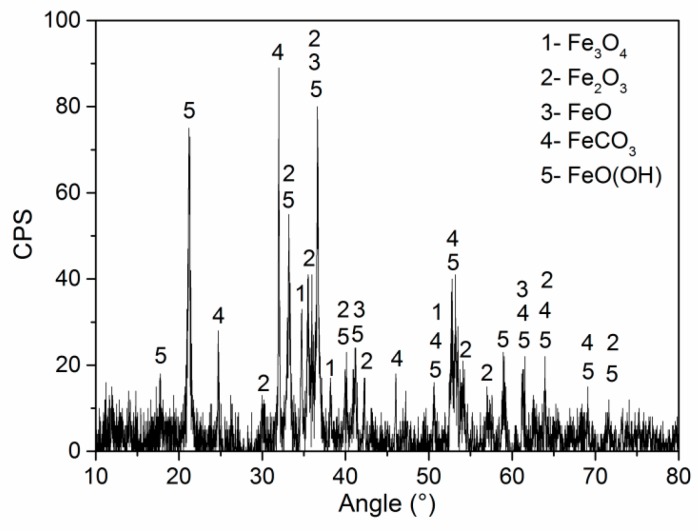
XRD analysis results of corrosion products.

The phase compositions of corrosion products on the 2″, 4″, and 6″ steel pipes were analyzed using XRD. The corrosion products on the surfaces of the three pipes are identical, and Figure 5 shows the XRD spectra of corrosion products from the 6″ steel pipe. The corrosion products are composed of Fe_3_O_4_, Fe_2_O_3_, FeO, FeCO_3_, and, FeO(OH). The counts per second (CPS) value in Figure 5 shows that FeO(OH) and FeCO_3_ have large shares in corrosion scale. Element S is found in the corrosion scale (see Figure 4). However, sulfide cannot be detected by XRD because of its low content; thus, sulfide has a minimal effect on the corrosion failure of steel pipes.

On the basis of the analysis results of EDS and XRD, the corrosion products on different pipes share the same chemical and phase compositions; however, only the 6″ pipe is perforated in the collected pipe sections. Given that the pipe materials and the transmission medium are the same, the only difference is the flow rate of the medium in the pipe. Under the given working conditions, the medium flow rate increases with the pipe diameter. Therefore, the influence of flow rate change on the microstructure of corrosion products is further observed.

**Figure 6:**
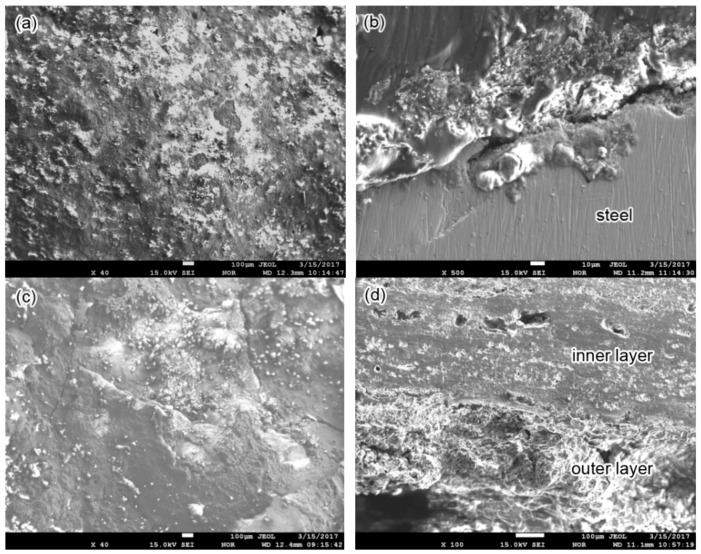

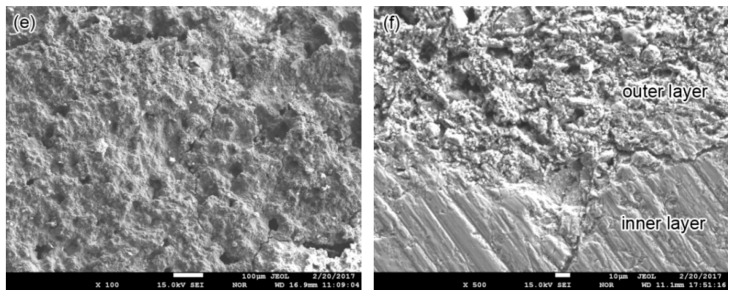
Top view (**a**,**c**,**e**) and cross-sectional view (**b**,**d**,**f**) of corrosion scales on steel pipes: (**a**,**b**) 2″ pipe, (**c**,**d**) 4″ pipe and (**e**,**f**) 6″ pipe.

The surface and cross-sectional morphology of the corrosion scale on the 2″, 4″ and 6″ steel pipes is shown in Figure 6. The scale on the 2″ pipe is dense (Figure 6a); however, the bond strength between the scale and steel substrate is weak, and cracking tends to occur at this interface (Figure 6b). The surface of scale on the 4″ pipe seems dense, and no pores can be found; however, cracks in scale are observed (Figure 6c). The cross-sectional observation also shows cracks and pores in the corrosion scale (Figure 6d), and the inner layer is stronger than the outer layer. The surface morphology of the corrosion scales on the 6″ pipe is shown in Figure 6e. Cracks and many pores are found in the corrosion scales; therefore, these scales can easily peel off. The cross-sectional morphology on the 6″ pipe is shown in Figure 6f, and the corrosion scales near the steel surface are denser than the outer parts. As shown in Figure 6, the corrosion products are porous at the beginning formation and will become denser with time. The dense inner layer can prevent penetration of the outer corrosive medium. However, the flow rate of the transmission medium will influence the structure of corrosion scale, and high flow rate can damage the integrity of the corrosion scale, thereby resulting in many pores and cracks in the scale. Intact and dense corrosion scales with strong adhesion can reduce the uniform corrosion rate, whereas defects and scale shedding can induce severe local corrosion [27,28]. The corrosive medium can penetrate along the paths formed by cracks and porosities [29], and corrosion can occur beneath corrosion products. Meanwhile, the peeling of the corrosion scale will lead to the exposure of fresh metal, thereby accelerating the corrosion here. Evidently, the 6″ pipe with loose corrosion products is most prone to corrosion failure.

### 3.3. Corrosion Simulation Test Results

#### 3.3.1. Failure Mode and Corrosion Products

**Figure 7:**
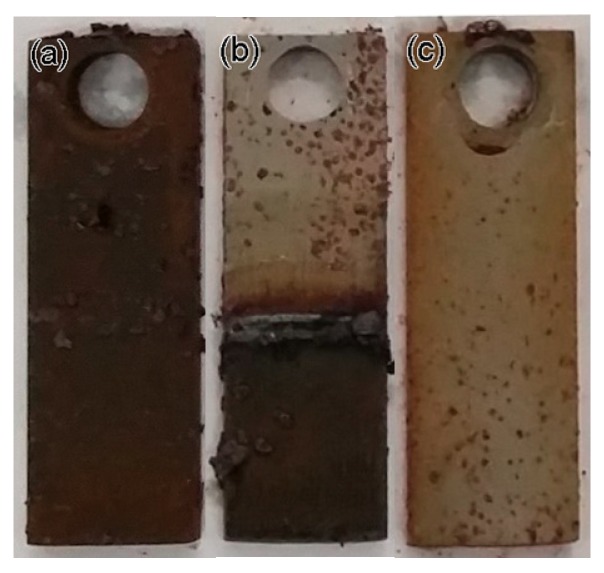
Morphology of specimens after corrosion test: (**a**) full immersion, (**b**) half immersion and (**c**) high-pressure gas exposure.

After 135 h in a simulated corrosion environment, the simulated gas was released from the kettle, and the specimens were removed. The specimens immersed in solution exhibits a general corrosion behavior, whereas the specimens exposed to gas presents a localized corrosion behavior. In comparison with other regions, more corrosion products are piled up near the waterline for the specimens partly immersed in the solution. Figure 7 shows each specimen in detail.

**Figure 8:**
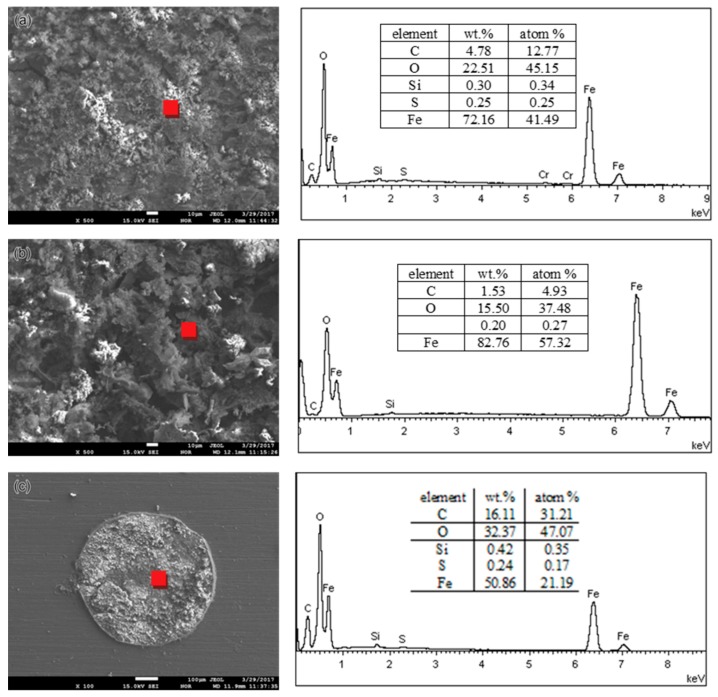
EDS analysis results of corrosion products: (**a**) full immersion, (**b**) half immersion and (**c**) high-pressure gas exposure.

**Figure 9:**
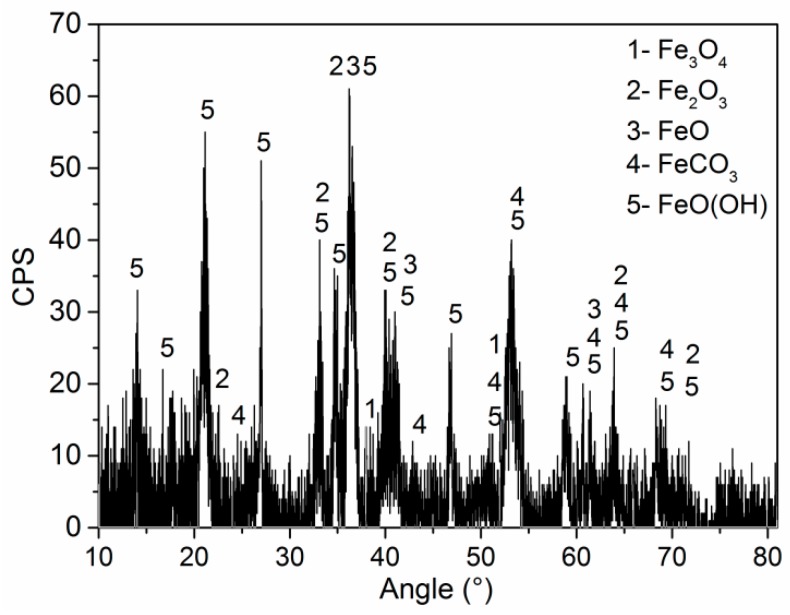
XRD spectra of corrosion products.

To compare the chemical and phase compositions of corrosion products obtained in the simulation tests with those collected from a leaking pipeline, the corroded specimens covered with corrosion products were investigated by EDS and XRD. Figure 8 shows the EDS analysis results. From the figure, the main elements include Fe and O for all the specimens, and the chemical compositions of corrosion products are the same as those of the corrosion products collected from the leaking pipeline (Figure 5). Figure 9 presents the XRD spectra of corrosion products, namely, Fe_3_O_4_, Fe_2_O_3_, FeO, FeCO_3_ and FeO(OH). FeO(OH) has the largest share in the corrosion scale.

#### 3.3.2. Corrosion Rate

**Figure 10:**
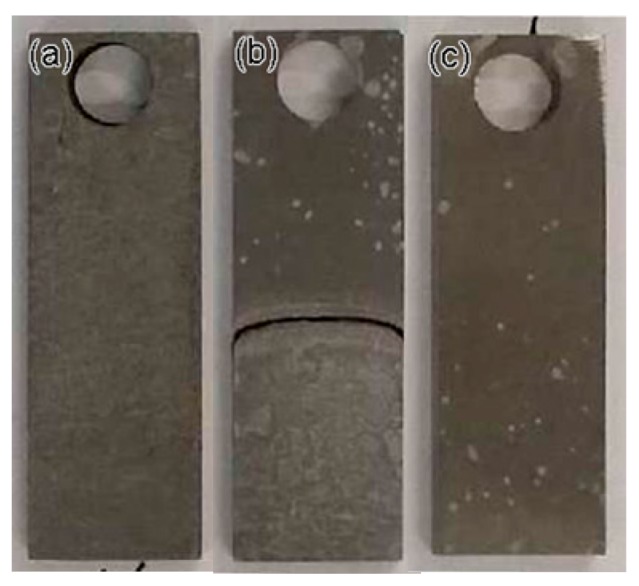
Morphology of specimens after removing corrosion products: (**a**) full immersion, (**b**) half immersion and (**c**) high-pressure gas exposure.

The specimens used for corrosion rate analysis were washed in an acid solution to remove the corrosion products. The acid solution was prepared following ISO 8407 using 500 mL of hydrochloric acid (HCl, *ρ* = 1.19 g/mL), 3.5 g of hexamethylenetetramine, and diluted to 1000 mL with distilled water. The specimens were then cleaned with ethanol and dried with argon gas stream, as shown in Figure 10. After removing the corrosion products, the specimens immersed in condensate water and those exposed to gas were corroded in a mode of general and localized corrosion, respectively. 

The corrosion rates with different corrosion failure modes were determined by using different approaches, as follows.

- General corrosion rate

The weight loss rate can be used to evaluate the general corrosion rate. The specimens before and after the corrosion test were weighed three times, and the relevant weight loss data are listed in Table 5. The weight loss rate was converted into a thickness loss rate to compare the general corrosion rate with the latter local corrosion rate. It is clear that the general corrosion rate of 2″ pipe is the slowest, while the corrosion rate of 4″and 6″ pipes is faster and substantially the same.

- Local corrosion rate in a gaseous environment

**Figure 11:**
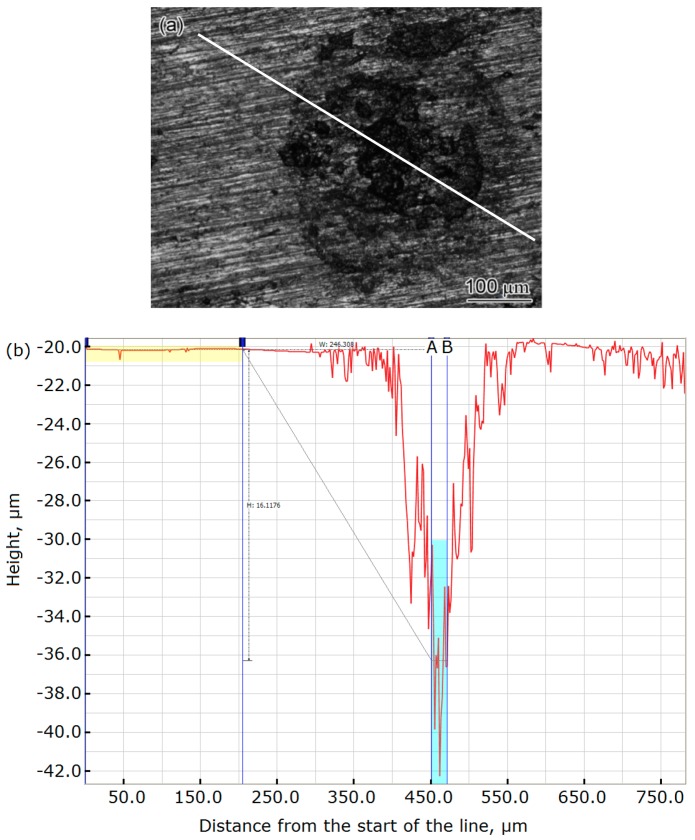
Surface morphology and profile of 2″ steel pipe specimen: (**a**) surface morphology and (**b**) profile along the line.

For the specimens exposed to a simulated transmission gas environment, the condensation of water droplets results in local corrosion. The depth of the corrosion pits was measured by a confocal microscope to determine the local corrosion rate. Figure 11a presents the surface morphology of the 2″ steel pipeline specimen, and the depth of the corrosion pit was measured based on the profile (Figure 11b) along the line in Figure 11a. The obtained 16.12 μm is the mean depth of the pit between lines A and B in Figure 11b. With this method, 10 corrosion pits were measured for the specimens from the 2″ pipe section. The average local corrosion rate of these pits and the highest local corrosion rate are listed in Table 6. The local corrosion rates for the specimens from the 4″ and 6″ pipe sections were also determined using the same method, and the results are also listed in Table 6. The 2″ pipe has the slowest corrosion rate, while the 4″and 6″ pipes have a faster and substantially identical corrosion rate. This is similar to the uniform corrosion rate of the three pipes in the full immersion zone, except that the corrosion rate of the material in the gas phase is slower than that in the full immersion zone.

- Uneven general corrosion near the waterline

**Figure 12:**
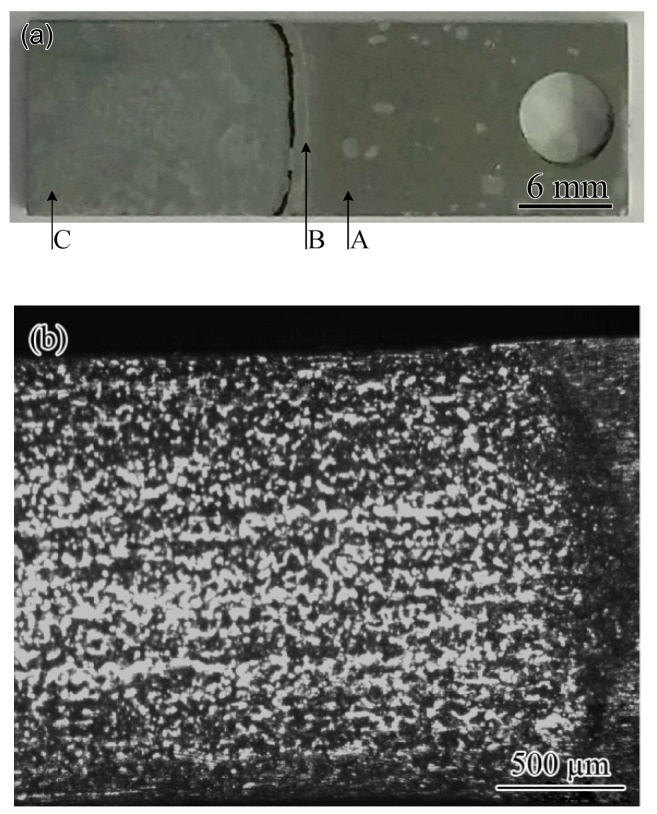
Morphology of specimen partly immersed in simulated condensate: (**a**) key positions, A—area with corrosion rate of 0 mm/y, B—waterline, C—area near the bottom; (**b**) change in thickness near the waterline.

General corrosion occurs below the waterline. However, the corrosion rate near the waterline is different from that far away from the waterline (Figure 12). The residual thicknesses in the areas exposed to gas, 1 mm below the waterline, 5 mm below the waterline, and near the bottom, were measured and recorded to determine the change rule of the corrosion rate near the waterline. Figure 12a shows the measuring positions. Here, the waterline is considered the critical position at which the overall thickness of the specimen begins to change. The region exposed to gas phase but without a corrosion pit was selected as a baseline, and the corrosion rate was set at 0 mm/y. Thus, the corrosion rate from the waterline to the bottom of the specimen can be determined, and the relevant data are listed in Table 7. For the areas from 5 mm below the waterline to the bottom of the specimen, the corrosion rate of the three specimens is approximately the same as the results in Table 5. This means that the area outside 5 mm below the waterline works in the same environment as the full immersion zone. Table 7 shows that the area 1 mm below the waterline exhibits a higher corrosion rate, which is approximately two times higher than that of the area outside 5 mm below the waterline. It is believed that the area in the range 5 mm below the waterline shows severe corrosion, especially the 1 mm area below the waterline.

## 4. Discussion

The corrosion products are composed of Fe_3_O_4_, Fe_2_O_3_, FeO, FeCO_3_ and FeO(OH). The CPS value in Figure 6 and Figure 9 show that FeO(OH) accounts for the largest proportion of corrosion products, followed by FeCO_3_; thus, the corrosion of steel pipe is controlled by oxygen absorption corrosion [30,31,32] and CO_2_ corrosion [33,34,35]. The presence of H_2_O is a necessary condition that produces FeO(OH) and FeCO_3_ [36]. H_2_O and O_2_ together with CO_2_ are the key factors that induce pipeline corrosion.

Oxygen absorption corrosion occurs when steel is exposed to an environment with water and oxygen [32].

Anodic reaction:

Fe − 2e^−^→Fe^2+^ (1)

Cathodic reaction:

O_2_ + 2H_2_O + 4e^−^→4OH^‒^ (2)

Fe^2+^ can be further oxidized to Fe^3+^:

4Fe^2+^ + O_2_→4Fe^3+^ + 2O^2−^ (3)

Then, the following multistep reactions and dehydration equilibria affect the formation of corrosion product.

Fe^2+^ + 2OH^−^→Fe(OH)_2_ (4)

Fe^3+^ + 3OH^−^→Fe(OH)_3_ (5)

Fe(OH)_2_→FeO + H_2_O (6)

Fe(OH)_3_→FeO(OH) + H_2_O (7)

2FeO(OH)→Fe_2_O_3_ + H_2_O (8)

With limited dissolved oxygen, Fe^2+^-containing materials, including FeO and Fe_3_O_4_, are favored. High oxygen concentrations favor the formation of Fe^3+^ materials [37]. Given that FeO(OH) has a large share in the corrosion scale, the oxygen concentration in the associated gas is high.

Oxygen concentration cell can be used to explain why the corrosion rate in the area of 1 mm below the waterline is the highest among different corrosion environments. Great variation in the oxygen concentration gradient occurs near the waterline [38]. The oxygen concentration decreases with water depth and finally reaches a stable value [39]. When the different areas of the same metal are in contact with a solution with different oxygen concentrations, the area with high oxygen concentration is good for the oxygen absorption reaction, whereas the metal dissolution reaction in this area is inhibited to a certain extent. By contrast, areas with low oxygen concentrations are subject to metal dissolution as anodes. It can be speculated from Figure 12 that the oxygen concentration changes within 1 mm below the waterline and then reaches a low stable value. The anodic reaction near the waterline is suppressed; thus, the degree of corrosion is lighter than that in large water depth. After leaving the waterline, the potential of the metal should not change after the oxygen concentration is stabilized. However, the area 1 mm below the waterline, where the oxygen concentration has only reached a stable low value, has a shorter conductive path than the areas farther from the waterline. Therefore, the anodic current concentrates flow through this narrow area and results in severe corrosion.

The mechanism of waterline corrosion can explain why corrosion perforation mostly occurs at the bottom of the actual gas pipeline. Figure 2 shows that numerous corrosion products are found at the bottom of the pipe. Notably, the amount of corrosion products in the gas pipeline indicates the degree of corrosion. The more products are present, the more severe the corrosion because although the wall shear stress caused by the gas flow in the pipeline can influence the structure of corrosion scale (Figure 4), this stress is insufficiently high to carry the corrosion product away [40]. Therefore, the corrosion product is easily deposited in the position where corrosion occurs. Figure 2 shows that the amount of corrosion products at the bottom of the pipe is considerably more than that in other areas, thereby indicating severe corrosion at the bottom of the pipe. On-site sampling proves the presence of condensate water at the bottom of the pipeline. When the depth of water is shallow, severe corrosion will occur at the bottom (Figure 2a). When the depth exceeds 5 mm, severe corrosion will occur at the symmetrical position on both sides of the bottom of the pipe (Figure 2b), and the severe corrosion sites correspond to the areas at a short distance below the waterline. Water with a depth of less than 5 mm is most likely to cause severe corrosion at the bottom of the pipe.

Corrosion of steel pipe in a gaseous environment only occurs in spots in which condensate water droplets exist. Therefore, local corrosion occurs. Table 5 and Table 6 show that the localized corrosion rate of the steel pipeline in a gaseous environment is slightly lower than the general corrosion rate of the specimen immersed in condensate. Analysis indicates two reasons for this situation. First, no autocatalysis of occluded corrosion cell occurs in the formation of a corrosion pit because the area around the pit without condensate cannot form a corrosion couple with the area with condensate droplets. Without electrolytes, no electrochemical reactions can occur. Localized corrosion in the gas phase is actually uniform corrosion that occurs at the precipitation location of condensate water. The other reason why the corrosion rate in a gaseous environment is lower than that in full immersion zones is that corrosion products are not easily washed away in gas, and the anodic process will be impeded by the formation of corrosion products.

The pH value of collected condensate water is 6.74; thus, acid gas corrosion should not be serious. Carbonic acid formation by CO_2_ dissolution and hydration is the major reaction during CO_2_ corrosion. At pH < 4, the hydrogen reduction is the dominant cathodic reaction, whereas direct reduction of carbonic acid becomes important at pH > 4 [41]. High pH results in a decreased solubility of iron carbonate and leads to an increased precipitation rate and high scaling tendency, and the higher pH of 6.6 results in the formation of several protective scales, which are reflected by a rapid decrease of the corrosion rate with time [42]. The pH value of the medium in the studied gas pipeline is as high as 6.74; however, corrosion perforation proves that the corrosion product does not have effective protection. This result also shows that CO_2_ does not play a leading role in the corrosion of the studied pipeline.

Generally, the dissolution of O_2_ and CO_2_ together with mineral ions let the condensate water be a strong corrosive medium, thereby resulting in steel pipe corrosion. The transport medium cannot carry the corrosion products away; hence, the corrosion products will increase with time, although their compactness is affected by the medium flow rate. Pores or cracks occur in the corrosion scale when the flow rate of the transmission medium is high (Figure 4e,f). The outer corrosive medium can penetrate through the corrosion scale along the paths formed by pores and cracks, thereby causing corrosion beneath the corrosion products. Furthermore, the oxygen concentration beneath the corrosion products is considerably lower than the open zones. The difference in oxygen concentration can accelerate the corrosion beneath the corrosion products, thereby promoting local corrosion.

H_2_O and O_2_ together with CO_2_ are the key factors that induce pipeline corrosion; thus, three measures can be taken to control the corrosion failure of gas pipeline, namely, dehydration, deoxygenation, and application of inhibitors. However, no standard is used to specify the oxygen content in natural gas, and deoxidization is not a common method in controlling gas pipeline corrosion. Here, we recommend deoxygenation and application of inhibitors. The corrosion rate of gas pipelines is directly related to the water content in natural gas [43]; therefore, strict dehydration is one of the most effective ways to control corrosion of gas transmission steel pipelines [44]. Given the lack of mature application experience of inhibitors at vapor/gas atmosphere as a reference, the injection of inhibitors can only be used as an auxiliary method.

## 5. Conclusions

(1) The corrosion products are mainly composed of FeO(OH) and FeCO_3_, and the corrosion of steel pipe is controlled by oxygen absorption corrosion and CO_2_ corrosion. Flow rates of the transmission medium affect the structure of the corrosion scale, and defects in scale promote the occurrence of medium penetration and local corrosion. 

(2) The specimens immersed in condensate are generally corroded, and the specimens exposed to a gaseous environment are locally corroded. The 1 mm area below the waterline has the highest corrosion rate, which is approximately two times higher than that of the fully immersed area far away from the waterline. Condensate with a depth of less than 5 mm is most likely to cause severe corrosion.

(3) The localized corrosion rate in a gaseous environment is slightly lower than the uniform corrosion rate in full immersion zone. Therefore, no autocatalysis of the occluded corrosion cell occurs in the formation of the corrosion pit, and a uniform corrosion essentially takes place in the precipitation location of the condensate water.

(4) The presence of water is a necessary condition for oxygen absorption corrosion and hydrogen evolution corrosion. Dehydration treatment of the transmission gas is suggested to be the main measure to control the corrosion failure of the associated gas pipeline.

## Figures and Tables

**Table 1:** Chemical composition of collected pipe materials and that for PSL 2 seamless X42 pipe in API Spec 5L-2013.

	C	Si	Mn	P	S	V	Nb	Ti	Cu	Ni	Cr	Mo
2″	0.167	0.245	0.408	0.009	0.025	0.000	0.000	0.000	0.018	0.000	0.027	0.000
4″	0.199	0.207	0.360	0.017	0.009	0.000	0.000	0.001	0.106	0.007	0.032	0.000
6″	0.175	0.251	0.499	0.010	0.010	0.000	0.000	0.000	0.100	0.000	0.036	0.000
Spec	≤0.24	≤0.40	≤1.20	≤0.025	≤0.015	≤0.06	≤0.05	≤0.04	≤0.50	≤0.30	≤0.30	≤0.15

Spec: acceptance criteria of chemical composition for PSL 2 seamless X42 pipe.

**Table 2:** Tensile results of collected steel pipes and acceptance criteria for PSL 2 X42 pipe.

Steel Sample	*R*_t0.5_ (MPa)	*R*_m_ (MPa)	*R*_t0.5_/*R*_m_
2″	435	500	0.87
4″	350	498	0.70
6″	375	515	0.73
Spec	290–495	415–655	≤0.93

*R*_t0.5_, yield strength (0.5 % total extension); *R*_m_, tensile strength; Spec: acceptance criteria of tensile properties for PSL 2 seamless X42 pipe.

**Table 3:** Compositions of transmission gas (vol.%).

Composition	Percent	Composition	Percent	Composition	Percent	Composition	Percent
Methane	72.72	Nitrogen	19.89	Ethane	3.66	CO_2_	1.30
Propane	0.87	Oxygen	0.47	n-Butane	0.36	i-Butane	0.25
iso-Pentane	0.19	N-Pentane	0.14	>C5	0.10	CO	0.05

**Table 4:** Ion concentration [C] of condensate water (mg/L).

Composition	[C]	Composition	[C]	Composition	[C]	Composition	[C]
Li^+^	0.45	Na^+^	24.18	NH_4_^+^	0.43	K^+^	2.3
Mg^2+^	0.14	Ca^2+^	1.62	Cl^-^	0.88	SO_4_^2-^	72.21

**Table 5:** Corrosion test results of full immersion.

Specimen	No.	Surface Area (mm^2^)	Weight Loss (g)	Corrosion Rate (mm/y)	
2″	1	1207.83	0.1637	1.12	1.15
	2	1211.13	0.1787	1.13	
	3	1208.51	0.1745	1.19	
4″	5	1332.04	0.2210	1.37	1.35
	6	1341.24	0.2203	1.36	
	7	1335.49	0.2117	1.31	
6″	9	1298.14	0.2198	1.40	1.34
	10	1350.60	0.2164	1.32	
	11	1333.63	0.2075	1.29	

**Table 6:** Localized corrosion rate for the three steel pipelines.

Specimen	Corrosion Pits Depth (μm)										Corrosion Rate (mm/y)	
	1	2	3	4	5	6	7	8	9	10	Ave.	Max.
2″	15.46	16.11	15.46	16.08	15.88	16.12	15.65	16.09	16.22	16.12	1.0330	1.0460
4″	19.36	19.64	18.98	19.17	19.55	18.87	19.86	19.52	18.64	19.06	1.2501	1.2744
6″	18.69	19.38	19.93	18.76	19.46	18.66	19.86	19.84	19.37	18.99	1.2520	1.2887

**Table 7:** Corrosion rate for specimens partly immersed in simulated condensate water (mm/y).

Specimen	Location		
	1 mm Below Waterline	5 mm Below Waterline	Near Bottom
2″	2.3661	1.2016	1.2146
4″	2.7675	1.3642	1.3578
6″	2.7864	1.3489	1.3562

## References

[1] Fan L., Zhang F. (2013). The study on effects of associated gas generations in oilfield grid. Environmental Protection and Resources Exploitation, PTS 1–3, Proceedings of International Conference on Advances in Energy and Environmental Science, Guangzhou, China, 30–31 July 2013.

[2] Lawal K.A., Ovuru M.I., Eyitayo S.I., Matemilola S., Adeniyi A.T. (2017). Underground storage as a solution for stranded associated gas in oil fields. J. Petrol. Sci. Eng..

[3] Moreno J., Badawy A., Kartoatmodjo G., AlShuraiqi H., Zulkhifly F., Tan L., Friedel T. (2009). Flaring, gas injection and reservoir management optimization: Preserving reservoir energy maximizes recovery and prolong the life of an ageing brown field. Proceedings of the Asia Pacific Oil and Gas Conference & Exhibition.

[4] Kaya E., Zarrouk S.J. (2017). Reinjection of greenhouse gases into geothermal reservoirs. Int. J. Greenh. Gas Con..

[5] Zheng D., Xu H., Wang J., Sun J., Zhao K., Li C., Shi L., Tang L. (2017). Key evaluation techniques in the process of gas reservoir being converted into underground gas storage. Petrol. Explor. Dev..

[6] Blann J.R., Laville G.M. (1995). Gas lifting a major oil field in Argentina with high CO_2_ content associated gas. Proceedings of the 1995 SPE Annual Technical Conference and Exhibition.

[7] Misener R., Gounaris C.E., Floudas C.A. (2009). Global optimization of gas lifting operations: A comparative study of piecewise linear formulations. Ind. Eng. Chem. Res..

[8] Javidi M., Bekhrad S. (2018). Failure analysis of a wet gas pipeline due to localised CO_2_ corrosion. Eng. Fail. Anal..

[9] Mansoori H., Mirzaee R., Esmaeilzadeh F., Vojood A., Dowrani A.S. (2017). Pitting corrosion failure analysis of a wet gas pipeline. Eng. Fail. Anal..

[10] Laumb J.D., Glazewski K.A., Hamling J.A., Azenkeng A., Kalenze N., Watson T.L. (2017). Corrosion and failure assessment for CO_2_ EOR and associated storage in the Weyburn Field. Eng. Procedia.

[11] Ilman M.N. (2014). Analysis of internal corrosion in subsea oil pipeline. Case Stud. Eng. Fail. Anal..

[12] Pouraria H., Seo J.K., Paik J.K. (2016). A numerical study on water wetting associated with the internal corrosion of oil pipelines. Ocean Eng..

[13] Ryder J., Tilekar J., Srinivasan S., Yap K.M. (2014). Advanced internal corrosion direct assessment methodology for wet gas/dry gas pipelines. Proceedings of the Corrosion 2014: Collaborate Educate Innovate Mitigate.

[14] Ren L., Jiang T., Jia Z.G., Li D.S., Yuan C.L., Li H.N. (2018). Pipeline corrosion and leakage monitoring based on the distributed optical fiber sensing technology. Measurement.

[15] Elhoud A., Jewilli F., Abouswa K., Rageai O. (2012). Failure analysis: Internal corrosion rupture of a 6-in gas line. Mater. Perform..

[16] Zhang H., Lan H.Q. (2017). A review of internal corrosion mechanism and experimental study for pipelines based on multiphase flow. Corros. Rev..

[17] Belarbi Z., Farelas F., Singer M., Nei S. (2016). Role of amines in the mitigation of CO_2_ top of the line corrosion. Corrosion.

[18] Ifezue D. (2017). Root cause failure analysis of corrosion in wet gas piping. J. Fail. Anal. Prev..

[19] Eckert R.B. (2012). Internal corrosion health check advised for liquid and gas pipeline operators. Mater. Perform..

[20] Wang B., Liu X.L., Xiong Z.X., Cheng J.C., Yang B., Yu C. (2018). Corrosion reasons and control measures of a natural gas pipeline. Surf. Technol..

[21] Qiao Q., Cheng G., Wu W., Li Y., Huang H., Wei Z. (2016). Failure analysis of corrosion at an inhomogeneous welded joint in a natural gas gathering pipeline considering the combined action of multiple factors. Eng. Fail. Anal..

[22] Choi Y.S., Nesic S., Young D. (2010). Effect of impurities on the corrosion behavior of CO_2_ transmission pipeline steel in supercritical CO_2_-water environments. Environ. Sci. Technol..

[23] Singer M., Al-Khamis J., Nesic S. (2013). Experimental study of sour top-of-the-line corrosion using a novel experimental setup. Corrosion.

[24] Singer M., Camacho A., Brown B., Neic S. (2011). Sour top-of-The-line corrosion in the presence of acetic acid. Corrosion.

[25] Kovalenko S.Y., Rybakov A.O., Klymenko A.V., Shytova L.H. (2012). Corrosion of the internal wall of a field gas pipeline. Mater. Sci..

[26] Wan H.X., Yang X.J., Liu Z.Y., Song D.D., Du C.W., Li X.G. (2017). Pitting behavior of L415 pipeline steel in simulated leaching liquid environment. J. Mater. Eng. Perform..

[27] Sun C., Sun J., Wang Y., Sui P., Lin X., Liu H., Cheng X., Zhou M. (2017). Effect of impurity interaction on the corrosion film characteristics and corrosion morphology evolution of X65 steel in water-saturated supercritical CO_2_ system. Int. J. Greenh. Gas Con..

[28] Sun C., Wang Y., Sun J., Lin X., Li X., Liu H., Cheng X. (2016). Effect of impurity on the corrosion behavior of X65 steel in water-saturated supercritical CO_2_ system. J. Supercrit. Fluid.

[29] Zhao W.-M., Wang Y., Dong L.-X., Wu K.-Y., Xue J. (2005). Corrosion mechanism of NiCrBSi coatings deposited by HVOF. Surf. Coat. Technol..

[30] De Oliveira L.A., Correa O.V., dos Santos D.J., Páez A.A.Z., de Oliveira M.C.L., Antunes R.A. (2018). Effect of silicate-based films on the corrosion behavior of the API 5L X80 pipeline steel. Corros. Sci..

[31] Wang S., Du C., Li X., Liu Z., Zhu M., Zhang D. (2015). Field corrosion characterization of soil corrosion of X70 pipeline steel in a red clay soil. Prog. Nat. Sci. Mater. Int..

[32] Liu H., Xu D., Dao A.Q., Zhang G., Lv Y., Liu H. (2015). Study of corrosion behavior and mechanism of carbon steel in the presence of Chlorella vulgaris. Corros. Sci..

[33] Yu J., Wang H., Yu Y., Luo Z., Liu W., Wang C. (2018). Corrosion behavior of X65 pipeline steel: Comparison of wet–Dry cycle and full immersion. Corros. Sci..

[34] De Motte R.A., Barker R., Burkle D., Vargas S.M., Neville A. (2018). The early stages of FeCO_3_ scale formation kinetics in CO_2_ corrosion. Mater. Chem. Phys..

[35] Ma Z., Yang Y., Brown B., Nesic S., Singer M. (2018). Investigation of precipitation kinetics of FeCO_3_ by EQCM. Corros. Sci..

[36] Pfennig A., Wolthusen H., Kranzmann A. (2017). Unusual corrosion behavior of 1.4542 exposed a laboratory saline aquifer water CCS-environment. Eng. Procedia.

[37] Speight J.G. (2014). Oil and Gas Corrosion Prevention: From Surface Facilities to Refineries.

[38] Wu J., Wang P., Gao J., Tan F., Zhang D., Cheng Y., Chen S. (2017). Comparison of water-line corrosion processes in natural and artificial seawater: The role of microbes. Electrochem. Commun..

[39] Jeffrey R., Melchers R.E. (2009). Corrosion of vertical mild steel strips in seawater. Corros. Sci..

[40] Li W., Pots B.F.M., Brown B., Kee K.E., Nesic S. (2016). A direct measurement of wall shear stress in multiphase flow-Is it an important parameter in CO_2_ corrosion of carbon steel pipelines?. Corros. Sci..

[41] Hatami S., Ghaderi-Ardakani A., Niknejad-Khomami M., Karimi-Malekabadi F., Rasaei M.R., Mohammadi A.H. (2016). On the prediction of CO_2_ corrosion in petroleum industry. J. Supercrit. Fluid.

[42] Nesic S. (2007). Key issues related to modelling of internal corrosion of oil and gas pipelines–A review. Corros. Sci..

[43] Kong Z.Y., Mahmoud A., Liu S., Sunarso J. (2018). Revamping existing glycol technologies in natural gas dehydration to improve the purity and absorption efficiency: Available methods and recent developments. J. Nat. Gas Sci. Eng..

[44] Sakheta A., Zahid U. (2018). Process simulation of dehydration unit for the comparative analysis of natural gas processing and carbon capture application. Chem. Eng. Res. Des..

